# μ-Propane-1,3-dithiol­ato-κ^4^ *S*,*S*′:*S*,*S*′-bis­[dicarbon­yl(triphenyl­phosphane-κ*P*)iron(II)](*Fe*—*Fe*)

**DOI:** 10.1107/S1600536811040621

**Published:** 2011-10-08

**Authors:** Bang-Shao Yin, Tian-Bao Li, Ming-Sheng Yang

**Affiliations:** a College of Chemistry and Chemical Engineering, Hunan Normal University, Hunan 410081, People’s Republic of China

## Abstract

The title compound, [Fe_2_(C_3_H_6_S_2_)(C_18_H_15_P)_2_(CO)_4_], which might serve as an active-site model of [FeFe]-hydrogenase, contains two fused Fe/S/C/C/C/S six-membered rings, one of which has a chair conformation and the other a boat conformation. Each Fe atom is coordinated by two carbonyl ligands, a triphenyl­phosphane ligand and a bis-bidentate dithiol­ate ligand, and also forms an Fe—Fe bond [2.5167 (16) Å]. Together, the six bonded atoms form a very distorted octa­hedral arrangement.

## Related literature

For details of the synthesis, see: Li *et al.* (2005 ▶). For more details about [FeFe]-hydrogenase model complexes, see: Song *et al.* (2005 ▶); Liu & Xiao (2011 ▶); Liu & Yin (2010 ▶, 2011 ▶); Liu *et al.* (2011 ▶).
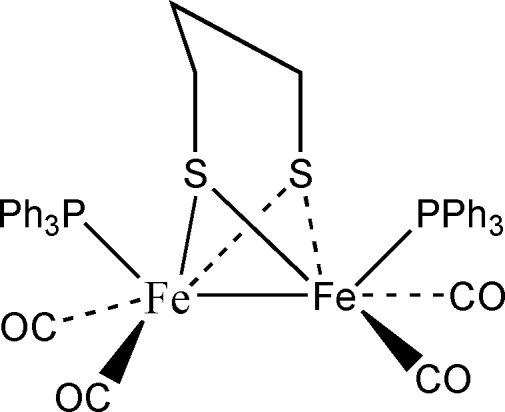


## Experimental

### 

#### Crystal data

[Fe_2_(C_3_H_6_S_2_)(C_18_H_15_P)_2_(CO)_4_]
*M* *_r_* = 854.48
Triclinic, 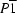

*a* = 9.139 (5) Å
*b* = 13.480 (5) Å
*c* = 16.786 (10) Å
α = 77.773 (19)°
β = 89.50 (2)°
γ = 71.187 (18)°
*V* = 1909.1 (17) Å^3^
*Z* = 2
Mo *K*α radiation
μ = 1.00 mm^−1^
*T* = 113 K
0.06 × 0.04 × 0.04 mm

#### Data collection

Rigaku Saturn724 CCD diffractometer
Absorption correction: multi-scan (*CrystalClear*; Rigaku/MSC, 2005 ▶) *T*_min_ = 0.943, *T*_max_ = 0.961
15996 measured reflections
6709 independent reflections
2718 reflections with *I* > 2σ(*I*)
*R*_int_ = 0.124

#### Refinement

*R*[*F* ^2^ > 2σ(*F* ^2^)] = 0.075
*wR*(*F* ^2^) = 0.146
*S* = 0.97
6709 reflections
478 parameters
H-atom parameters constrained
Δρ_max_ = 1.35 e Å^−3^
Δρ_min_ = −0.66 e Å^−3^

### 

Data collection: *CrystalClear* (Rigaku/MSC, 2005 ▶); cell refinement: *CrystalClear*; data reduction: *CrystalClear*; program(s) used to solve structure: *SHELXS97* (Sheldrick, 2008 ▶); program(s) used to refine structure: *SHELXL97* (Sheldrick, 2008 ▶); molecular graphics: *XP* in *SHELXTL* (Sheldrick, 2008 ▶); software used to prepare material for publication: *CrystalStructure* (Rigaku/MSC, 2005 ▶).

## Supplementary Material

Crystal structure: contains datablock(s) global, I. DOI: 10.1107/S1600536811040621/hb6432sup1.cif

Structure factors: contains datablock(s) I. DOI: 10.1107/S1600536811040621/hb6432Isup2.hkl

Additional supplementary materials:  crystallographic information; 3D view; checkCIF report

## Figures and Tables

**Table 1:** Selected bond lengths (Å)

Fe1—C2	1.719 (8)
Fe1—C1	1.773 (8)
Fe1—P1	2.237 (2)
Fe1—S2	2.254 (2)
Fe1—S1	2.285 (2)
Fe2—C4	1.720 (8)
Fe2—C3	1.750 (8)
Fe2—P2	2.230 (2)
Fe2—S1	2.276 (2)
Fe2—S2	2.287 (2)

**Fig. 1.**
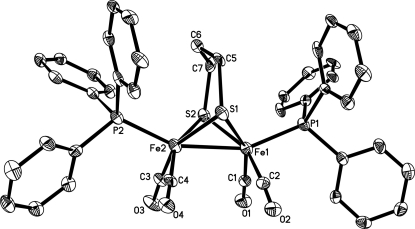
The molecular structure of (I). Displacement ellipsoids are drawn at the 30% probability level and H atoms are shown as small spheres of arbitrary radii.

**Fig. 2.**
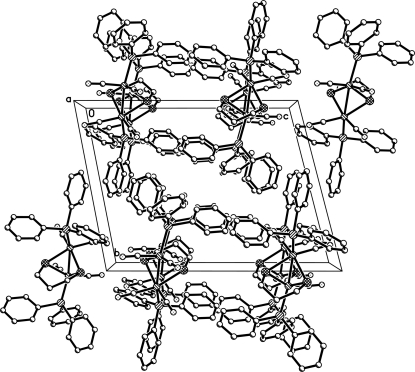
The crystal packing for (I).

## supplementary crystallographic information

### Comment

[FeFe]-hydrogenases are a class of natural enzymes that can catalyze the production and consumption of hydrogen gas in several microorganisms (Song *et al.*, 2005); Liu & Yin, 2010, 2011); Liu & Xiao, 2011; Liu *et al.*, 2011). In continuated our work, a [FeFe]-hydrogenases model complex had been synthesized. The strucuture was confirmed by X-ray crstallography.

Single-crystal X-ray diffraction analysis reveals that the title complex crystallizes in the triclinic space group P-1. As shown in Fig. 1, the title complex contains four carbonyls and two PPh_3_ ligands. The diiron propanedithiolate consists of two fused six-membered rings, in which one ring has a chair conformation and the other ring has a boat conformation. The PPh_3_ ligands reside in an axial position of the square-pyramidal geometry of the Fe atoms. As shown in Fig. 2, the crystal structure is stabilized by van der Waals interactions.

### Experimental

The title complex was prepared according to the literature procedures (Li *et al* , 2005)). Crystals were grown from slow evaporation of dichloromethane and hexane solution at room temperature.

### Refinement

All the H atoms were positioned geometrically (C—H = 0.93–0.97 Å) and refined as riding with *U*_iso_(H) = 1.2*U*_eq_(C) or 1.5*U*_eq_(methyl C).

### Crystal data

**Table:**

[Fe_2_(C_3_H_6_S_2_)(C_18_H_15_P)_2_(CO)_4_]	*Z* = 2
*M**_r_* = 854.48	*F*(000) = 880
Triclinic, *P*1	*D*_x_ = 1.486 Mg m^−^^3^
*a* = 9.139 (5) Å	Mo *K*α radiation, λ = 0.71073 Å
*b* = 13.480 (5) Å	Cell parameters from 5985 reflections
*c* = 16.786 (10) Å	θ = 1.6–26.1°
α = 77.773 (19)°	µ = 1.00 mm^−^^1^
β = 89.50 (2)°	*T* = 113 K
γ = 71.187 (18)°	Prism, colorless
*V* = 1909.1 (17) Å^3^	0.06 × 0.04 × 0.04 mm

### Data collection

**Table:**

Rigaku Saturn724 CCD diffractometer	6709 independent reflections
Radiation source: rotating anode	2718 reflections with *I* > 2σ(*I*)
multilayer	*R*_int_ = 0.124
Detector resolution: 14.22 pixels mm^-1^	θ_max_ = 25.0°, θ_min_ = 1.6°
ω and φ scans	*h* = −10→10
Absorption correction: multi-scan (*CrystalClear*; Rigaku/MSC, 2005)	*k* = −16→12
*T*_min_ = 0.943, *T*_max_ = 0.961	*l* = −19→19
15996 measured reflections	

### Refinement

**Table:**

Refinement on *F*^2^	Primary atom site location: structure-invariant direct methods
Least-squares matrix: full	Secondary atom site location: difference Fourier map
*R*[*F*^2^ > 2σ(*F*^2^)] = 0.075	Hydrogen site location: inferred from neighbouring sites
*wR*(*F*^2^) = 0.146	H-atom parameters constrained
*S* = 0.97	*w* = 1/[σ^2^(*F*_o_^2^) + (0.0041*P*)^2^] where *P* = (*F*_o_^2^ + 2*F*_c_^2^)/3
6709 reflections	(Δ/σ)_max_ = 0.001
478 parameters	Δρ_max_ = 1.35 e Å^−^^3^
0 restraints	Δρ_min_ = −0.66 e Å^−^^3^

### Special details

**Table:**

Geometry. All e.s.d.'s (except the e.s.d. in the dihedral angle between two l.s. planes) are estimated using the full covariance matrix. The cell e.s.d.'s are taken into account individually in the estimation of e.s.d.'s in distances, angles and torsion angles; correlations between e.s.d.'s in cell parameters are only used when they are defined by crystal symmetry. An approximate (isotropic) treatment of cell e.s.d.'s is used for estimating e.s.d.'s involving l.s. planes.
Refinement. Refinement of *F*^2^ against ALL reflections. The weighted *R*-factor *wR* and goodness of fit *S* are based on *F*^2^, conventional *R*-factors *R* are based on *F*, with *F* set to zero for negative *F*^2^. The threshold expression of *F*^2^ > σ(*F*^2^) is used only for calculating *R*-factors(gt) *etc*. and is not relevant to the choice of reflections for refinement. *R*-factors based on *F*^2^ are statistically about twice as large as those based on *F*, and *R*- factors based on ALL data will be even larger.

### Fractional atomic coordinates and isotropic or equivalent isotropic displacement parameters (Å^2^)

**Table:**

	*x*	*y*	*z*	*U*_iso_*/*U*_eq_	
Fe1	0.87669 (12)	1.09597 (7)	0.19524 (6)	0.0333 (3)	
Fe2	1.07650 (12)	0.91292 (7)	0.24157 (6)	0.0341 (3)	
P1	0.6388 (2)	1.21710 (14)	0.18129 (12)	0.0316 (5)	
P2	1.1691 (2)	0.74053 (13)	0.30622 (12)	0.0314 (5)	
S1	0.9131 (2)	0.99887 (13)	0.32702 (11)	0.0359 (5)	
S2	0.8517 (2)	0.94621 (13)	0.16626 (12)	0.0356 (5)	
O1	0.9526 (7)	1.1484 (4)	0.0249 (3)	0.0531 (16)	
O2	1.0428 (6)	1.2238 (4)	0.2488 (3)	0.0423 (14)	
O3	1.2386 (6)	0.9101 (4)	0.0911 (3)	0.0574 (17)	
O4	1.3209 (6)	0.9648 (4)	0.3122 (3)	0.0482 (15)	
C1	0.9215 (8)	1.1291 (5)	0.0921 (5)	0.035 (2)	
C2	0.9700 (9)	1.1748 (5)	0.2264 (4)	0.037 (2)	
C3	1.1720 (8)	0.9076 (5)	0.1509 (5)	0.040 (2)	
C4	1.2219 (9)	0.9448 (5)	0.2834 (4)	0.0342 (19)	
C5	0.7706 (8)	0.9347 (5)	0.3615 (4)	0.038 (2)	
H5A	0.8029	0.8936	0.4183	0.045*	
H5B	0.6712	0.9921	0.3632	0.045*	
C6	0.7389 (8)	0.8584 (5)	0.3127 (5)	0.039 (2)	
H6A	0.8317	0.7935	0.3190	0.047*	
H6B	0.6523	0.8353	0.3362	0.047*	
C7	0.6992 (8)	0.9069 (5)	0.2232 (5)	0.040 (2)	
H7A	0.6056	0.9712	0.2172	0.049*	
H7B	0.6725	0.8545	0.1978	0.049*	
C8	0.6245 (8)	1.3568 (5)	0.1372 (4)	0.0308 (18)	
C9	0.7434 (9)	1.3833 (5)	0.0962 (5)	0.037 (2)	
H9	0.8371	1.3271	0.0938	0.045*	
C10	0.7330 (9)	1.4864 (5)	0.0589 (4)	0.042 (2)	
H10	0.8180	1.5017	0.0323	0.051*	
C11	0.5929 (9)	1.5688 (5)	0.0612 (4)	0.039 (2)	
H11	0.5804	1.6408	0.0343	0.047*	
C12	0.4745 (9)	1.5446 (6)	0.1024 (5)	0.043 (2)	
H12	0.3808	1.6006	0.1051	0.051*	
C13	0.4893 (9)	1.4402 (5)	0.1400 (4)	0.044 (2)	
H13	0.4055	1.4253	0.1682	0.052*	
C14	0.4933 (8)	1.2007 (5)	0.1161 (5)	0.0325 (19)	
C15	0.5438 (8)	1.1447 (5)	0.0520 (4)	0.036 (2)	
H15	0.6512	1.1110	0.0473	0.043*	
C16	0.4372 (8)	1.1397 (5)	−0.0023 (4)	0.0322 (18)	
H16	0.4730	1.1051	−0.0459	0.039*	
C17	0.2815 (9)	1.1824 (5)	0.0038 (5)	0.042 (2)	
H17	0.2095	1.1747	−0.0330	0.050*	
C18	0.2319 (9)	1.2374 (5)	0.0653 (5)	0.041 (2)	
H18	0.1240	1.2693	0.0701	0.049*	
C19	0.3360 (8)	1.2466 (5)	0.1195 (5)	0.040 (2)	
H19	0.2983	1.2857	0.1605	0.048*	
C20	0.5427 (8)	1.2287 (5)	0.2775 (4)	0.0261 (17)	
C21	0.5778 (9)	1.2937 (5)	0.3258 (5)	0.043 (2)	
H21	0.6423	1.3355	0.3065	0.051*	
C22	0.5154 (9)	1.2951 (6)	0.4027 (5)	0.044 (2)	
H22	0.5350	1.3399	0.4350	0.053*	
C23	0.4285 (9)	1.2335 (6)	0.4304 (5)	0.044 (2)	
H23	0.3891	1.2346	0.4829	0.053*	
C24	0.3951 (8)	1.1701 (5)	0.3862 (5)	0.042 (2)	
H24	0.3334	1.1268	0.4073	0.051*	
C25	0.4529 (9)	1.1688 (5)	0.3083 (5)	0.041 (2)	
H25	0.4284	1.1250	0.2766	0.049*	
C26	1.1264 (8)	0.6384 (5)	0.2631 (4)	0.0314 (18)	
C27	1.0331 (9)	0.6666 (5)	0.1930 (5)	0.037 (2)	
H27	0.9904	0.7404	0.1671	0.045*	
C28	0.9989 (8)	0.5915 (5)	0.1585 (5)	0.039 (2)	
H28	0.9361	0.6139	0.1090	0.047*	
C29	1.0557 (8)	0.4852 (5)	0.1959 (5)	0.037 (2)	
H29	1.0286	0.4332	0.1744	0.044*	
C30	1.1545 (8)	0.4541 (5)	0.2664 (4)	0.037 (2)	
H30	1.1975	0.3801	0.2917	0.044*	
C31	1.1907 (8)	0.5291 (5)	0.2998 (4)	0.0325 (19)	
H31	1.2587	0.5067	0.3474	0.039*	
C32	1.1094 (8)	0.7174 (5)	0.4129 (4)	0.0304 (19)	
C33	1.0258 (8)	0.6510 (5)	0.4409 (4)	0.0336 (19)	
H33	1.0012	0.6101	0.4066	0.040*	
C34	0.9755 (8)	0.6434 (5)	0.5219 (5)	0.037 (2)	
H34	0.9205	0.5955	0.5428	0.044*	
C35	1.0078 (9)	0.7065 (5)	0.5695 (4)	0.040 (2)	
H35	0.9710	0.7038	0.6227	0.048*	
C36	1.0909 (8)	0.7721 (6)	0.5418 (5)	0.041 (2)	
H36	1.1132	0.8140	0.5760	0.049*	
C37	1.1437 (8)	0.7786 (5)	0.4640 (5)	0.037 (2)	
H37	1.2029	0.8243	0.4450	0.044*	
C38	1.3813 (8)	0.6816 (5)	0.3197 (4)	0.0302 (18)	
C39	1.4600 (9)	0.6203 (5)	0.3913 (5)	0.044 (2)	
H39	1.4042	0.6103	0.4387	0.053*	
C40	1.6209 (10)	0.5719 (6)	0.3968 (5)	0.051 (2)	
H40	1.6742	0.5324	0.4478	0.061*	
C41	1.7028 (9)	0.5819 (5)	0.3263 (5)	0.047 (2)	
H41	1.8116	0.5475	0.3283	0.056*	
C42	1.6247 (9)	0.6408 (5)	0.2565 (5)	0.042 (2)	
H42	1.6801	0.6487	0.2087	0.050*	
C43	1.4680 (9)	0.6905 (5)	0.2509 (5)	0.038 (2)	
H43	1.4172	0.7316	0.1996	0.045*	

### Atomic displacement parameters (Å^2^)

**Table:**

	*U*^11^	*U*^22^	*U*^33^	*U*^12^	*U*^13^	*U*^23^
Fe1	0.0382 (7)	0.0381 (6)	0.0270 (7)	−0.0102 (5)	0.0135 (5)	−0.0182 (5)
Fe2	0.0353 (7)	0.0404 (6)	0.0284 (7)	−0.0078 (5)	0.0130 (5)	−0.0191 (5)
P1	0.0332 (12)	0.0366 (10)	0.0252 (12)	−0.0072 (9)	0.0094 (10)	−0.0142 (9)
P2	0.0324 (12)	0.0381 (10)	0.0254 (12)	−0.0074 (9)	0.0083 (9)	−0.0177 (9)
S1	0.0384 (12)	0.0419 (11)	0.0294 (12)	−0.0086 (9)	0.0137 (10)	−0.0201 (9)
S2	0.0384 (12)	0.0385 (10)	0.0311 (13)	−0.0080 (9)	0.0100 (10)	−0.0181 (9)
O1	0.062 (4)	0.068 (4)	0.036 (4)	−0.026 (3)	0.024 (3)	−0.020 (3)
O2	0.050 (4)	0.054 (3)	0.033 (3)	−0.024 (3)	0.014 (3)	−0.022 (3)
O3	0.044 (4)	0.099 (4)	0.033 (4)	−0.020 (3)	0.023 (3)	−0.028 (3)
O4	0.039 (4)	0.056 (3)	0.049 (4)	−0.014 (3)	0.009 (3)	−0.013 (3)
C1	0.033 (5)	0.034 (4)	0.044 (6)	−0.014 (4)	0.005 (4)	−0.014 (4)
C2	0.045 (5)	0.037 (4)	0.026 (5)	−0.013 (4)	0.019 (4)	−0.005 (4)
C3	0.028 (5)	0.049 (5)	0.042 (6)	−0.010 (4)	0.008 (4)	−0.016 (4)
C4	0.030 (5)	0.042 (4)	0.028 (5)	−0.007 (4)	0.017 (4)	−0.009 (4)
C5	0.031 (4)	0.041 (4)	0.029 (5)	0.002 (4)	0.020 (4)	−0.006 (4)
C6	0.033 (5)	0.035 (4)	0.045 (6)	−0.006 (4)	0.010 (4)	−0.007 (4)
C7	0.048 (5)	0.022 (4)	0.057 (6)	−0.010 (4)	0.005 (5)	−0.023 (4)
C8	0.033 (5)	0.036 (4)	0.025 (5)	−0.009 (4)	0.005 (4)	−0.016 (3)
C9	0.040 (5)	0.030 (4)	0.044 (6)	−0.010 (4)	0.012 (4)	−0.016 (4)
C10	0.045 (5)	0.057 (5)	0.034 (5)	−0.024 (4)	0.015 (4)	−0.018 (4)
C11	0.056 (6)	0.034 (4)	0.026 (5)	−0.014 (4)	0.003 (4)	0.000 (4)
C12	0.049 (6)	0.045 (5)	0.030 (5)	−0.005 (4)	−0.001 (4)	−0.017 (4)
C13	0.048 (6)	0.041 (4)	0.037 (5)	−0.005 (4)	0.014 (4)	−0.013 (4)
C14	0.034 (5)	0.029 (4)	0.038 (5)	−0.011 (3)	0.009 (4)	−0.012 (3)
C15	0.032 (5)	0.038 (4)	0.030 (5)	0.002 (4)	0.007 (4)	−0.016 (4)
C16	0.034 (5)	0.037 (4)	0.025 (5)	−0.007 (4)	0.012 (4)	−0.015 (3)
C17	0.042 (5)	0.049 (5)	0.044 (6)	−0.020 (4)	0.002 (4)	−0.023 (4)
C18	0.029 (5)	0.064 (5)	0.043 (6)	−0.023 (4)	0.020 (4)	−0.029 (4)
C19	0.036 (5)	0.043 (4)	0.054 (6)	−0.018 (4)	0.025 (4)	−0.032 (4)
C20	0.026 (4)	0.026 (4)	0.027 (5)	−0.008 (3)	0.009 (4)	−0.009 (3)
C21	0.050 (5)	0.041 (4)	0.038 (6)	−0.012 (4)	0.016 (4)	−0.017 (4)
C22	0.043 (5)	0.063 (5)	0.032 (5)	−0.014 (4)	0.008 (4)	−0.029 (4)
C23	0.045 (6)	0.055 (5)	0.023 (5)	−0.003 (4)	0.012 (4)	−0.013 (4)
C24	0.038 (5)	0.036 (4)	0.038 (6)	0.002 (4)	0.021 (4)	−0.001 (4)
C25	0.044 (5)	0.035 (4)	0.029 (5)	0.009 (4)	0.006 (4)	−0.012 (4)
C26	0.035 (5)	0.034 (4)	0.027 (5)	−0.007 (4)	0.022 (4)	−0.019 (3)
C27	0.043 (5)	0.032 (4)	0.038 (5)	−0.006 (4)	0.016 (4)	−0.021 (4)
C28	0.037 (5)	0.046 (4)	0.040 (5)	−0.013 (4)	0.013 (4)	−0.023 (4)
C29	0.038 (5)	0.036 (4)	0.044 (6)	−0.013 (4)	0.018 (4)	−0.022 (4)
C30	0.040 (5)	0.035 (4)	0.032 (5)	−0.002 (4)	0.015 (4)	−0.017 (4)
C31	0.028 (4)	0.049 (4)	0.024 (5)	−0.010 (4)	0.008 (4)	−0.021 (4)
C32	0.019 (4)	0.035 (4)	0.033 (5)	0.003 (3)	−0.002 (4)	−0.016 (4)
C33	0.030 (5)	0.033 (4)	0.032 (5)	0.000 (4)	0.003 (4)	−0.013 (4)
C34	0.034 (5)	0.033 (4)	0.034 (5)	−0.001 (3)	0.017 (4)	−0.004 (4)
C35	0.043 (5)	0.055 (5)	0.022 (5)	−0.011 (4)	0.018 (4)	−0.017 (4)
C36	0.037 (5)	0.060 (5)	0.031 (5)	−0.011 (4)	0.016 (4)	−0.027 (4)
C37	0.034 (5)	0.038 (4)	0.037 (5)	−0.005 (4)	0.003 (4)	−0.016 (4)
C38	0.041 (5)	0.032 (4)	0.020 (5)	−0.014 (4)	0.013 (4)	−0.009 (3)
C39	0.036 (5)	0.053 (5)	0.043 (6)	−0.012 (4)	0.014 (4)	−0.015 (4)
C40	0.050 (6)	0.063 (5)	0.037 (6)	−0.008 (5)	−0.004 (5)	−0.019 (4)
C41	0.037 (5)	0.050 (5)	0.052 (6)	−0.002 (4)	0.014 (5)	−0.028 (5)
C42	0.043 (5)	0.050 (5)	0.033 (5)	−0.013 (4)	0.014 (4)	−0.015 (4)
C43	0.042 (5)	0.045 (4)	0.023 (5)	−0.009 (4)	0.002 (4)	−0.010 (4)

### Geometric parameters (Å, °)

**Table:**

Fe1—C2	1.719 (8)	C17—H17	0.9500
Fe1—C1	1.773 (8)	C18—C19	1.374 (9)
Fe1—P1	2.237 (2)	C18—H18	0.9500
Fe1—S2	2.254 (2)	C19—H19	0.9500
Fe1—S1	2.285 (2)	C20—C25	1.355 (9)
Fe1—Fe2	2.5167 (16)	C20—C21	1.419 (9)
Fe2—C4	1.720 (8)	C21—C22	1.408 (9)
Fe2—C3	1.750 (8)	C21—H21	0.9500
Fe2—P2	2.230 (2)	C22—C23	1.339 (10)
Fe2—S1	2.276 (2)	C22—H22	0.9500
Fe2—S2	2.287 (2)	C23—C24	1.349 (9)
P1—C14	1.826 (7)	C23—H23	0.9500
P1—C8	1.834 (7)	C24—C25	1.407 (9)
P1—C20	1.843 (7)	C24—H24	0.9500
P2—C26	1.829 (7)	C25—H25	0.9500
P2—C38	1.839 (7)	C26—C27	1.369 (9)
P2—C32	1.859 (8)	C26—C31	1.399 (8)
S1—C5	1.812 (7)	C27—C28	1.382 (8)
S2—C7	1.837 (8)	C27—H27	0.9500
O1—C1	1.156 (8)	C28—C29	1.365 (8)
O2—C2	1.189 (8)	C28—H28	0.9500
O3—C3	1.171 (9)	C29—C30	1.398 (9)
O4—C4	1.160 (8)	C29—H29	0.9500
C5—C6	1.538 (8)	C30—C31	1.380 (8)
C5—H5A	0.9900	C30—H30	0.9500
C5—H5B	0.9900	C31—H31	0.9500
C6—C7	1.502 (9)	C32—C33	1.366 (9)
C6—H6A	0.9900	C32—C37	1.409 (9)
C6—H6B	0.9900	C33—C34	1.425 (9)
C7—H7A	0.9900	C33—H33	0.9500
C7—H7B	0.9900	C34—C35	1.380 (9)
C8—C9	1.384 (9)	C34—H34	0.9500
C8—C13	1.385 (9)	C35—C36	1.352 (9)
C9—C10	1.372 (8)	C35—H35	0.9500
C9—H9	0.9500	C36—C37	1.384 (9)
C10—C11	1.405 (9)	C36—H36	0.9500
C10—H10	0.9500	C37—H37	0.9500
C11—C12	1.369 (10)	C38—C39	1.362 (9)
C11—H11	0.9500	C38—C43	1.402 (9)
C12—C13	1.378 (9)	C39—C40	1.398 (10)
C12—H12	0.9500	C39—H39	0.9500
C13—H13	0.9500	C40—C41	1.400 (10)
C14—C19	1.376 (9)	C40—H40	0.9500
C14—C15	1.432 (9)	C41—C42	1.329 (10)
C15—C16	1.367 (9)	C41—H41	0.9500
C15—H15	0.9500	C42—C43	1.366 (9)
C16—C17	1.364 (9)	C42—H42	0.9500
C16—H16	0.9500	C43—H43	0.9500
C17—C18	1.387 (9)		
			
C2—Fe1—C1	93.2 (3)	C19—C14—P1	124.3 (6)
C2—Fe1—P1	96.3 (2)	C15—C14—P1	118.7 (6)
C1—Fe1—P1	96.4 (2)	C16—C15—C14	119.9 (7)
C2—Fe1—S2	156.9 (2)	C16—C15—H15	120.0
C1—Fe1—S2	86.5 (2)	C14—C15—H15	120.0
P1—Fe1—S2	106.71 (9)	C17—C16—C15	122.5 (7)
C2—Fe1—S1	87.4 (2)	C17—C16—H16	118.7
C1—Fe1—S1	156.3 (2)	C15—C16—H16	118.7
P1—Fe1—S1	107.06 (8)	C16—C17—C18	117.8 (7)
S2—Fe1—S1	83.84 (8)	C16—C17—H17	121.1
C2—Fe1—Fe2	100.6 (2)	C18—C17—H17	121.1
C1—Fe1—Fe2	100.5 (2)	C19—C18—C17	121.1 (7)
P1—Fe1—Fe2	155.34 (8)	C19—C18—H18	119.4
S2—Fe1—Fe2	56.98 (6)	C17—C18—H18	119.4
S1—Fe1—Fe2	56.33 (6)	C18—C19—C14	121.8 (7)
C4—Fe2—C3	90.2 (3)	C18—C19—H19	119.1
C4—Fe2—P2	93.3 (2)	C14—C19—H19	119.1
C3—Fe2—P2	100.2 (2)	C25—C20—C21	118.4 (7)
C4—Fe2—S1	88.2 (2)	C25—C20—P1	122.5 (5)
C3—Fe2—S1	153.7 (2)	C21—C20—P1	118.7 (6)
P2—Fe2—S1	106.10 (9)	C22—C21—C20	118.8 (7)
C4—Fe2—S2	156.0 (2)	C22—C21—H21	120.6
C3—Fe2—S2	87.6 (3)	C20—C21—H21	120.6
P2—Fe2—S2	110.61 (8)	C23—C22—C21	120.3 (7)
S1—Fe2—S2	83.30 (8)	C23—C22—H22	119.8
C4—Fe2—Fe1	101.1 (2)	C21—C22—H22	119.8
C3—Fe2—Fe1	98.0 (2)	C22—C23—C24	122.0 (8)
P2—Fe2—Fe1	156.70 (8)	C22—C23—H23	119.0
S1—Fe2—Fe1	56.69 (6)	C24—C23—H23	119.0
S2—Fe2—Fe1	55.71 (6)	C23—C24—C25	119.0 (8)
C14—P1—C8	100.7 (3)	C23—C24—H24	120.5
C14—P1—C20	103.1 (3)	C25—C24—H24	120.5
C8—P1—C20	102.0 (3)	C20—C25—C24	121.5 (7)
C14—P1—Fe1	117.7 (2)	C20—C25—H25	119.3
C8—P1—Fe1	116.2 (2)	C24—C25—H25	119.3
C20—P1—Fe1	114.9 (2)	C27—C26—C31	118.1 (6)
C26—P2—C38	99.4 (3)	C27—C26—P2	120.9 (5)
C26—P2—C32	104.5 (3)	C31—C26—P2	121.0 (6)
C38—P2—C32	102.6 (3)	C26—C27—C28	122.5 (6)
C26—P2—Fe2	119.9 (2)	C26—C27—H27	118.8
C38—P2—Fe2	115.9 (2)	C28—C27—H27	118.8
C32—P2—Fe2	112.5 (2)	C29—C28—C27	119.8 (7)
C5—S1—Fe2	112.2 (2)	C29—C28—H28	120.1
C5—S1—Fe1	116.5 (2)	C27—C28—H28	120.1
Fe2—S1—Fe1	66.98 (7)	C28—C29—C30	118.8 (6)
C7—S2—Fe1	111.5 (2)	C28—C29—H29	120.6
C7—S2—Fe2	115.3 (2)	C30—C29—H29	120.6
Fe1—S2—Fe2	67.31 (7)	C31—C30—C29	121.2 (6)
O1—C1—Fe1	178.5 (6)	C31—C30—H30	119.4
O2—C2—Fe1	175.9 (6)	C29—C30—H30	119.4
O3—C3—Fe2	175.3 (7)	C30—C31—C26	119.6 (7)
O4—C4—Fe2	179.0 (7)	C30—C31—H31	120.2
C6—C5—S1	118.7 (5)	C26—C31—H31	120.2
C6—C5—H5A	107.6	C33—C32—C37	119.7 (7)
S1—C5—H5A	107.6	C33—C32—P2	123.1 (5)
C6—C5—H5B	107.6	C37—C32—P2	117.0 (6)
S1—C5—H5B	107.6	C32—C33—C34	119.6 (7)
H5A—C5—H5B	107.1	C32—C33—H33	120.2
C7—C6—C5	114.1 (5)	C34—C33—H33	120.2
C7—C6—H6A	108.7	C35—C34—C33	119.1 (7)
C5—C6—H6A	108.7	C35—C34—H34	120.5
C7—C6—H6B	108.7	C33—C34—H34	120.5
C5—C6—H6B	108.7	C36—C35—C34	121.3 (7)
H6A—C6—H6B	107.6	C36—C35—H35	119.4
C6—C7—S2	115.6 (5)	C34—C35—H35	119.4
C6—C7—H7A	108.4	C35—C36—C37	120.3 (7)
S2—C7—H7A	108.4	C35—C36—H36	119.8
C6—C7—H7B	108.4	C37—C36—H36	119.8
S2—C7—H7B	108.4	C36—C37—C32	119.9 (7)
H7A—C7—H7B	107.4	C36—C37—H37	120.0
C9—C8—C13	117.2 (7)	C32—C37—H37	120.0
C9—C8—P1	122.0 (5)	C39—C38—C43	117.0 (7)
C13—C8—P1	120.7 (6)	C39—C38—P2	124.0 (6)
C10—C9—C8	123.4 (7)	C43—C38—P2	118.6 (6)
C10—C9—H9	118.3	C38—C39—C40	121.6 (8)
C8—C9—H9	118.3	C38—C39—H39	119.2
C9—C10—C11	118.0 (7)	C40—C39—H39	119.2
C9—C10—H10	121.0	C39—C40—C41	119.5 (8)
C11—C10—H10	121.0	C39—C40—H40	120.3
C12—C11—C10	119.6 (7)	C41—C40—H40	120.3
C12—C11—H11	120.2	C42—C41—C40	118.5 (8)
C10—C11—H11	120.2	C42—C41—H41	120.8
C11—C12—C13	121.0 (7)	C40—C41—H41	120.8
C11—C12—H12	119.5	C41—C42—C43	122.6 (8)
C13—C12—H12	119.5	C41—C42—H42	118.7
C12—C13—C8	120.9 (8)	C43—C42—H42	118.7
C12—C13—H13	119.6	C42—C43—C38	120.9 (7)
C8—C13—H13	119.6	C42—C43—H43	119.6
C19—C14—C15	116.8 (7)	C38—C43—H43	119.6
			
C2—Fe1—Fe2—C4	−0.7 (3)	Fe1—S1—C5—C6	55.4 (5)
C1—Fe1—Fe2—C4	94.6 (3)	S1—C5—C6—C7	−53.4 (8)
P1—Fe1—Fe2—C4	−133.1 (3)	C5—C6—C7—S2	62.7 (7)
S2—Fe1—Fe2—C4	173.4 (3)	Fe1—S2—C7—C6	−73.7 (5)
S1—Fe1—Fe2—C4	−80.4 (3)	Fe2—S2—C7—C6	0.5 (5)
C2—Fe1—Fe2—C3	−92.5 (4)	C14—P1—C8—C9	−112.5 (7)
C1—Fe1—Fe2—C3	2.8 (3)	C20—P1—C8—C9	141.5 (6)
P1—Fe1—Fe2—C3	135.1 (3)	Fe1—P1—C8—C9	15.8 (7)
S2—Fe1—Fe2—C3	81.6 (3)	C14—P1—C8—C13	63.9 (6)
S1—Fe1—Fe2—C3	−172.2 (3)	C20—P1—C8—C13	−42.1 (6)
C2—Fe1—Fe2—P2	126.3 (3)	Fe1—P1—C8—C13	−167.8 (5)
C1—Fe1—Fe2—P2	−138.4 (3)	C13—C8—C9—C10	−0.1 (11)
P1—Fe1—Fe2—P2	−6.0 (3)	P1—C8—C9—C10	176.4 (5)
S2—Fe1—Fe2—P2	−59.59 (19)	C8—C9—C10—C11	−1.4 (11)
S1—Fe1—Fe2—P2	46.63 (18)	C9—C10—C11—C12	2.2 (11)
C2—Fe1—Fe2—S1	79.7 (3)	C10—C11—C12—C13	−1.6 (12)
C1—Fe1—Fe2—S1	175.0 (2)	C11—C12—C13—C8	0.1 (12)
P1—Fe1—Fe2—S1	−52.67 (18)	C9—C8—C13—C12	0.7 (11)
S2—Fe1—Fe2—S1	−106.21 (10)	P1—C8—C13—C12	−175.8 (6)
C2—Fe1—Fe2—S2	−174.1 (3)	C8—P1—C14—C19	−72.6 (7)
C1—Fe1—Fe2—S2	−78.8 (2)	C20—P1—C14—C19	32.5 (7)
P1—Fe1—Fe2—S2	53.54 (18)	Fe1—P1—C14—C19	160.1 (5)
S1—Fe1—Fe2—S2	106.21 (10)	C8—P1—C14—C15	101.6 (6)
C2—Fe1—P1—C14	159.2 (4)	C20—P1—C14—C15	−153.3 (5)
C1—Fe1—P1—C14	65.3 (4)	Fe1—P1—C14—C15	−25.7 (7)
S2—Fe1—P1—C14	−23.0 (3)	C19—C14—C15—C16	0.6 (11)
S1—Fe1—P1—C14	−111.6 (3)	P1—C14—C15—C16	−174.0 (5)
Fe2—Fe1—P1—C14	−67.8 (3)	C14—C15—C16—C17	−3.0 (11)
C2—Fe1—P1—C8	39.7 (4)	C15—C16—C17—C18	3.4 (11)
C1—Fe1—P1—C8	−54.2 (3)	C16—C17—C18—C19	−1.4 (12)
S2—Fe1—P1—C8	−142.5 (3)	C17—C18—C19—C14	−0.9 (12)
S1—Fe1—P1—C8	129.0 (3)	C15—C14—C19—C18	1.3 (11)
Fe2—Fe1—P1—C8	172.8 (3)	P1—C14—C19—C18	175.6 (6)
C2—Fe1—P1—C20	−79.1 (3)	C14—P1—C20—C25	39.2 (6)
C1—Fe1—P1—C20	−173.1 (3)	C8—P1—C20—C25	143.4 (6)
S2—Fe1—P1—C20	98.7 (3)	Fe1—P1—C20—C25	−90.1 (6)
S1—Fe1—P1—C20	10.1 (3)	C14—P1—C20—C21	−148.0 (5)
Fe2—Fe1—P1—C20	53.9 (3)	C8—P1—C20—C21	−43.9 (6)
C4—Fe2—P2—C26	−154.4 (4)	Fe1—P1—C20—C21	82.7 (5)
C3—Fe2—P2—C26	−63.6 (4)	C25—C20—C21—C22	−1.3 (10)
S1—Fe2—P2—C26	116.5 (3)	P1—C20—C21—C22	−174.4 (5)
S2—Fe2—P2—C26	27.7 (3)	C20—C21—C22—C23	2.0 (11)
Fe1—Fe2—P2—C26	77.3 (3)	C21—C22—C23—C24	−1.2 (12)
C4—Fe2—P2—C38	−35.2 (3)	C22—C23—C24—C25	−0.2 (11)
C3—Fe2—P2—C38	55.6 (4)	C21—C20—C25—C24	−0.1 (10)
S1—Fe2—P2—C38	−124.3 (3)	P1—C20—C25—C24	172.7 (5)
S2—Fe2—P2—C38	146.9 (3)	C23—C24—C25—C20	0.9 (10)
Fe1—Fe2—P2—C38	−163.6 (3)	C38—P2—C26—C27	−129.7 (6)
C4—Fe2—P2—C32	82.3 (3)	C32—P2—C26—C27	124.6 (6)
C3—Fe2—P2—C32	173.1 (4)	Fe2—P2—C26—C27	−2.5 (7)
S1—Fe2—P2—C32	−6.8 (3)	C38—P2—C26—C31	48.9 (6)
S2—Fe2—P2—C32	−95.5 (3)	C32—P2—C26—C31	−56.8 (6)
Fe1—Fe2—P2—C32	−46.0 (3)	Fe2—P2—C26—C31	176.1 (5)
C4—Fe2—S1—C5	−145.0 (3)	C31—C26—C27—C28	1.1 (11)
C3—Fe2—S1—C5	128.1 (6)	P2—C26—C27—C28	179.7 (5)
P2—Fe2—S1—C5	−52.1 (3)	C26—C27—C28—C29	1.8 (11)
S2—Fe2—S1—C5	57.5 (3)	C27—C28—C29—C30	−3.4 (11)
Fe1—Fe2—S1—C5	110.5 (3)	C28—C29—C30—C31	2.2 (11)
C4—Fe2—S1—Fe1	104.5 (2)	C29—C30—C31—C26	0.6 (11)
C3—Fe2—S1—Fe1	17.6 (6)	C27—C26—C31—C30	−2.2 (10)
P2—Fe2—S1—Fe1	−162.59 (8)	P2—C26—C31—C30	179.2 (5)
S2—Fe2—S1—Fe1	−53.01 (7)	C26—P2—C32—C33	−11.0 (6)
C2—Fe1—S1—C5	151.2 (3)	C38—P2—C32—C33	−114.3 (5)
C1—Fe1—S1—C5	−116.7 (6)	Fe2—P2—C32—C33	120.5 (5)
P1—Fe1—S1—C5	55.4 (2)	C26—P2—C32—C37	173.8 (5)
S2—Fe1—S1—C5	−50.2 (2)	C38—P2—C32—C37	70.6 (5)
Fe2—Fe1—S1—C5	−104.3 (2)	Fe2—P2—C32—C37	−54.6 (5)
C2—Fe1—S1—Fe2	−104.5 (2)	C37—C32—C33—C34	−0.7 (9)
C1—Fe1—S1—Fe2	−12.4 (6)	P2—C32—C33—C34	−175.7 (4)
P1—Fe1—S1—Fe2	159.70 (8)	C32—C33—C34—C35	2.3 (9)
S2—Fe1—S1—Fe2	54.07 (7)	C33—C34—C35—C36	−2.5 (10)
C2—Fe1—S2—C7	124.4 (7)	C34—C35—C36—C37	1.0 (11)
C1—Fe1—S2—C7	−145.6 (3)	C35—C36—C37—C32	0.7 (10)
P1—Fe1—S2—C7	−50.0 (3)	C33—C32—C37—C36	−0.8 (9)
S1—Fe1—S2—C7	56.0 (3)	P2—C32—C37—C36	174.5 (5)
Fe2—Fe1—S2—C7	109.5 (3)	C26—P2—C38—C39	−96.6 (6)
C2—Fe1—S2—Fe2	14.9 (6)	C32—P2—C38—C39	10.7 (6)
C1—Fe1—S2—Fe2	104.9 (2)	Fe2—P2—C38—C39	133.6 (5)
P1—Fe1—S2—Fe2	−159.49 (8)	C26—P2—C38—C43	76.7 (6)
S1—Fe1—S2—Fe2	−53.49 (7)	C32—P2—C38—C43	−176.1 (5)
C4—Fe2—S2—C7	−120.2 (6)	Fe2—P2—C38—C43	−53.2 (6)
C3—Fe2—S2—C7	154.6 (3)	C43—C38—C39—C40	2.5 (10)
P2—Fe2—S2—C7	54.6 (2)	P2—C38—C39—C40	175.8 (5)
S1—Fe2—S2—C7	−50.1 (2)	C38—C39—C40—C41	−3.2 (11)
Fe1—Fe2—S2—C7	−104.0 (2)	C39—C40—C41—C42	2.3 (11)
C4—Fe2—S2—Fe1	−16.2 (6)	C40—C41—C42—C43	−0.9 (12)
C3—Fe2—S2—Fe1	−101.4 (2)	C41—C42—C43—C38	0.2 (11)
P2—Fe2—S2—Fe1	158.63 (8)	C39—C38—C43—C42	−1.0 (10)
S1—Fe2—S2—Fe1	53.90 (7)	P2—C38—C43—C42	−174.7 (5)
Fe2—S1—C5—C6	−19.0 (6)		
